# A New Fossil Species of *Nothotsuga* from the Mula Basin, Litang County, Sichuan Province and Its Paleoclimate and Paleoecology Significance

**DOI:** 10.3390/biology12010046

**Published:** 2022-12-26

**Authors:** Junling Dong, Zhe Li, Jingxin Gao, Qian Wang, Bainian Sun

**Affiliations:** 1 State Key Laboratory of Oil and Gas Reservoir Geology and Exploitation, Institute of Sedimentary Geology, Chengdu University of Technology, Chengdu 610059, China; 2 School of Earth Sciences and Key Laboratory of Mineral Resources in Western China (Gansu Province), Lanzhou University, Lanzhou 730000, China; 3 State Key Laboratory of Paleobiology and Stratigraphy, Nanjing Institute of Geology and Paleontology, Chinese Academy of Sciences, Nanjing 210008, China

**Keywords:** paleoclimate, paleoecology, *Nothotsuga*, Mula Basin, Litang County

## Abstract

**Simple Summary:**

In this study, we describe the well-preserved fossil seed cones of *Nothotsuga* found in the Mula Basin, Litang County, Sichuan Province, southwestern China. By detailed morphological comparisons, a new fossil species, *Nothotsuga mulaensis* Z. Li et J. L. Dong sp. nov. is named. This discovery suggests that *Nothotsuga* had a more northern distribution during the Miocene. In addition, it suggests that the Mula region had a humid and warm climate during this period. The ancient landform presents an intermountain lake basin, which would have been surrounded by higher mountains. After the Miocene, due to the uplift of the Tibetan Plateau, *Nothotsuga* was either unable to adapt to the extinction of the local environmental or was able to migrate to several scattered places in southern China.

**Abstract:**

In this paper, we describe a new fossil species, *Nothotsuga mulaensis* Z. Li & J.L. Dong sp. nov. The discovery of the fossil species was based on well-preserved fossil seed cones that were found in the Mula Basin in Xiamula village, Litang County, Sichuan Province, southwestern China. The shapes of these fossils were characterized by ovate seed cones, rhombic or suborbicular scales with auriculate bases, and the bracts were ligulate-spathulate in shape. This finding suggests that *Nothotsuga* once had a wide distribution range in China and that it also inhabited the eastern Tibetan Plateau (TP). *Nothotsuga mulaensis* was distributed in an intermountain lake basin, at altitudes from 2000 to 2300 m, in a warm and humid environment. This finding also suggests that the eastern TP may have provided good habitat for *Nothotsuga* during the Miocene. In addition, we propose that the uplift, accompanied by the severe cooling and aridification that occurred after the Miocene, caused the disappearance of this species of *Nothotsuga* in the eastern TP.

## 1. Introduction

The uplift of the Tibetan Plateau (TP) dramatically changed the regional topography and climate, which profoundly impacted the evolution and distribution of plants, and it promoted either diversification or extinction [1,2]. The Hengduan Mountains region (HDM) is one of the world’s biodiversity hotspots. It is characterized by high species richness and endemism due to the complex topography and climate conditions that exist there [3,4]. Understanding the evolution of biodiversity can provide valuable information for the evolution of plants and the conservation of endemic species [5,6]. However, the biodiversity of the Hengduan Mountains regions is still unexplored due to the lack of fossil records.

*Nothotsuga* Hu ex C. N. Page is a monotypic genus of Pinaceae, contains a single species, *N. longibracteata* (W.C. Cheng) Hu ex C. N. Page, which is endemic to the mountainous regions of mid-subtropical China, and is restricted to 24°49′–28°34′N, 107°56′–118°21′E [7]. The systematic position of this genus was previously the focus of much debate and was, initially, placed in the genus *Tsuga* (Endl.) Carr. [7,8], and later treated as a separate genus due to the exposed spathulate bracts of its seed cones [8]. *Nothotsuga longibracteata* prefers a warm, humid, cloudy, and foggy environment, and an altitude gradient of 300−2300 m [7,9,10,11,12,13]. It is usually found sporadically scattered in evergreen and deciduous broad-leaved forests or mixed in broad-leaved forests on upper slopes, ridges, and peaks to form small patches of unique montane mixed forests [12]. Thus, *Nothotsuga* is of great significance in understanding the paleoclimate and paleoecology.

*Nothotsuga* is a relict “living fossil plant”. Furthermore, it is an economically important genus due to its valuable timber and medicinal applications [12]. It has a long evolutionary history, which dates back to the late Eocene of Baltic amber [14] and it was diverse during the Miocene [15,16,17,18]. However, recent studies have shown that *Nothotsuga longibracteata* is vulnerable to global climate change and human activities and is, thus, limited to a narrow range [8]. It is endangered and treated as a grade three state-protected plant, which is included in the *Red Book of Chinese Plants* [19] and *the Red List of Chinese Species* [20].

In this paper, we present the three-dimensional, well-preserved fossil cones of *Nothotsuga*, which were retrieved from the Miocene Mula Basin of Xiamula village, Litang County, Sichuan Province, China. Through a detailed comparative analysis, we discovered a new fossil species, *Nothotsuga mulaensis* Z. Li & J. L. Dong sp. nov. Furthermore, in this paper we discuss the biogeographic significance and the paleoclimate and paleoecology of the Mula Basin during the Miocene, based on the modern ecological tolerances of *Nothotsuga*. This study provides a comprehensive understanding of the evolutionary history of the eastern TP’s biodiversity, and of the paleoenvironment and paleoecology of the eastern TP.

## 2. Geological Backgrounds

**Figure 1:**
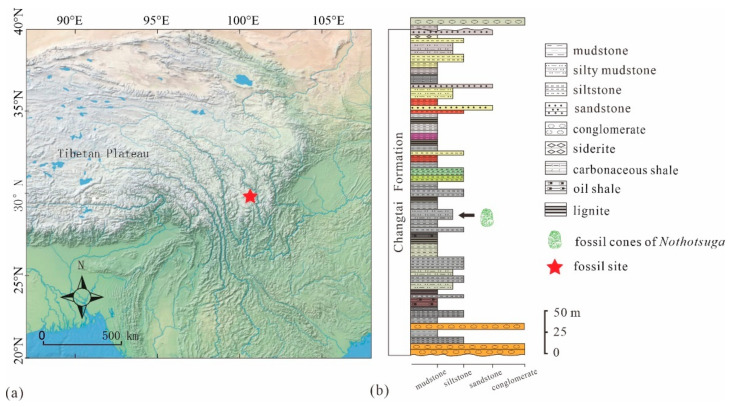
(**a**) The location of fossil site in Xiamula village, Litang County, western Sichuan Province. (**b**) Stratigraphic sequence of the Miocene Changtai Formation.

The well-preserved fossil seed cones of *Nothotsuga* were found in the Mula Basin, in the Ganzi Autonomous Prefecture, eastern Sichuan Province, southwestern China (Figure 1a). The Mula Basin is an intermountain basin, or a graben basin, and is located in the southern Yidun Terrane. Furthermore, it is a flexural basin that formed in an alluvial environment and developed in a far-field, upper-crustal response to the plateau outgrowth. This plateau outgrowth resulted from the India–Asia collision, which occurred prior to the onset of the dominant strike-slip deformation throughout the eastern TP [21]. Based on the U-Pb zircon geochronology and thermochronology results, this is considered an Oligocene age for the development of the Mula Basin [21,22].

The Mula Basin is well known for its Cenozoic strata. Based on the lithostratigraphy and biostratigraphy, these strata are composed of a lower Mula Formation and an upper Jiawa Formation. However, the Mula Formation has now been deactivated because it is synonymous with the Changtai Formation [23,24]; therefore, we have used the Changtai Formation instead of the Mula Formation in this paper. The Changtai Formation is characterized by a series of coal seams, mainly coal-containing strata and coal-containing deposits, which mainly consist of sandstone, mudstone with conglomerate, lignite formation, and thin siderite (Figure 1b) [25].

Since no volcanic material or mammals have been found in the Changtai Formation, the age of the strata sequence-estimations which has been based on fossil and pollen evidence, has long been disputed and remains unclear. While the plant fossil assemblages indicate that the age of the strata sequence ranges from the Pliocene to the Pleistocene [25,26], it is also considered to predate to the Pliocene, as reported in the palynological records [23]. Alternatively, other views hold that the deposition period of the Changtai Formation is contemporaneous with the Maladun Formation, which was regarded as dating back to the early middle Miocene [23,27]. Furthermore, the fossil gastropods suggest that it dates back to the Miocene [25]. In this paper, we hold that the Changtai Formation of the Mula Basin was deposited in the Miocene, which may well be the early middle Miocene.

Fossil seed cones of *Nothotsuga* were obtained from the north side of the road approximately 1 km southeast of Xiamula village, which is located along the Bangong-Nujiang Suture (BNS) (29.57° N, 100.69° E). The fossiliferous layer is in the lower to middle part of the Changtai Formation, which is characterized by argillaceous siltstone and indicates an ancient lacustrine or a swamp environment [24].

## 3. Materials and Methods

In this study, we describe four three-dimensional, well-preserved fossil cones of *Nothotsuga* in gray mudstone and siltstone. Several well-preserved fossils from the mudstone have already been briefly described in other papers, such as *Trapa*, *Tsuga*, *Abies*, *Picea*, and *Spiraea* [26]. A palynological analysis of the mudstone, siltstone, and lignite showed that a mixed deciduous broad-leaved forest, and a coniferous forest were present in the nearby highlands, which was dominated by *Picea*, *Tsuga*, *Ericipites*, *Betula*, and *Compositae* [24].

We attempted to remove the residual sediment covering on the surface of the seed cones using an ultrasonic instrument to obtain clear specimens. Next, the specimens were observed using a stereomicroscope (LEICA M165C stereomicroscope) and, for a general view, an alpha 6000 digital camera with an SEL30M35, was used. Measurements were made from the photographs using ImageJ software.

The living species for comparative analysis were downloaded from the Chinese Plant Image Library (http://ppbc.iplant.cn/about, 25 April 2022) and from Royal Botanic Garden, Edinburgh (https:/data.rbge.org.uk, 5 March 2022). The morphological data of the extant species were from the Flora of China [28] and the gymnosperm database [29]. Terms were obtained from Ding et al. [17] and Wu et al. [30]. These cones were housed in the herbarium of the Nature Museum in the Chengdu University of Technology.

## 4. Systematic Paleontology

**Figure 2:**
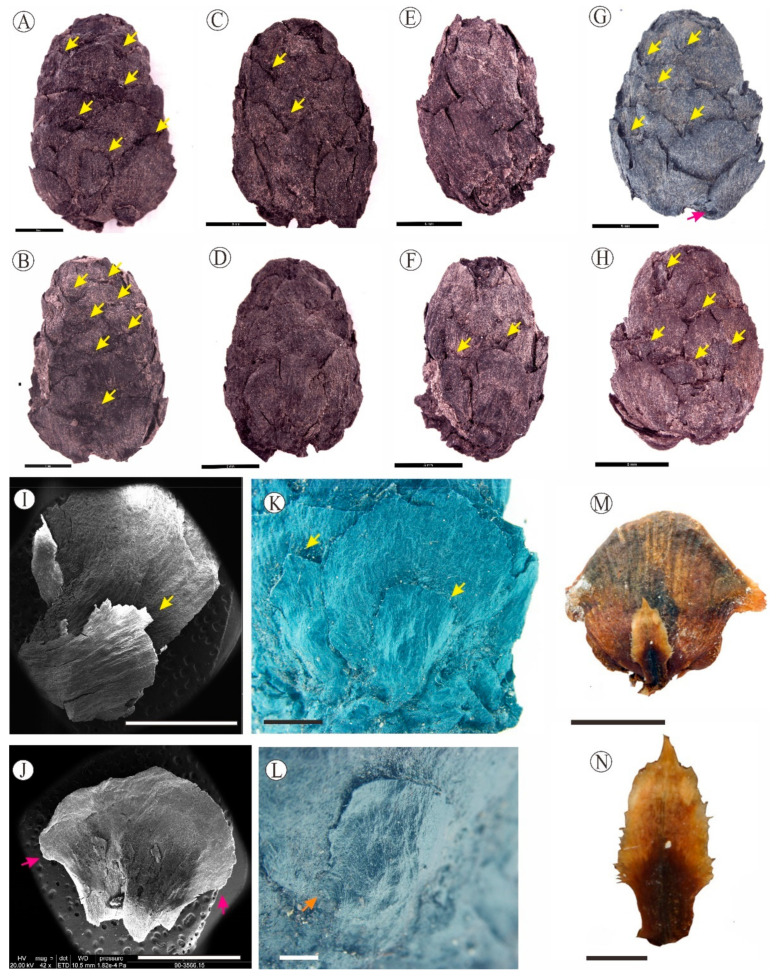
(**A**–**L**) *Nothotsuga mulaensis* from the Mula Basin, Litang County, Sichuan Province and (**M**,**N**) Seed scale and bract of *N. longibracteata*, which are from Xu et al. [31]. (**A**–**H**) Seed cones as seen from both sides, showing the exserted bracts with yellow arrows and auriculate scales with pink arrows. (**I**) Seed scale with bract of *N. mulaensis* under the scanning electron microscope, showing the bract with yellow arrows. (**J**) Scale of *N. mulaensis* under the scanning electron microscope, showing the auriculate bases with pink arrows. (**K**) Seed scale with bract in abaxial view, showing acute or acuminate bract with yellow arrows. (**L**) Bract of *N. mulaensis*, showing ligulate-spathulate in shape. (**M**) Seed scale with bract in abaxial view of *N. longibracteata*, showing auriculate scale. (**N**) Bract of *N. longibracteata*, showing dentate margin and acute apex. (**A**–**H**) Scale bar = 5 mm; (**I**,**J**,**N**) Scale bar = 3 mm; (**K**) Scale bar = 2 mm; (**L**) Scale bar = 1 mm; (**M**) Scale bar = 8 mm.

Order: Pinales Dumortier
Family: Pinaceae Spreng. ex F. Rudolphi
Genus: *Nothotsuga* Hu ex C. N. Page
Species: *Nothotsuga mulaensis* Z. Li & J. L. Dong, sp. nov.
Holotype: LTML001 (Figure 2A,B)
Paratypes: LTML002 (Figure 2C,D), LTML003 (Figure 2E,F), LTML004 (Figure 2G,H)
Type locality: Xiamula village, Litang County, Sichuan Province, southwestern China.
Type horizon: Changtai Formation, Miocene.
Repository: Nature Museum of the Chengdu University of Technology.
Etymology: The specific epithet “*mula*” refers to the fossil site, Mula region, Litang County, the eastern Tibetan Plateau.

### 4.1. Diagnosis

Fossil seed cones ovate, apex round to acute, base broadly rounded; ovuliferous scales spirally arranged, rhombic to suborbicular, texture; abaxially broad, semicircular at the upper margin, contracted into a small denticulate tip; basally small, lateral to the base with two wing-like extensions; scales rhombic to suborbicular, rounded apex and auriculate bases. Bract scales ligulate-spathulate and exserted, 1/2 to 2/3 as long as seed scales, apex acute or acuminate, with dentate margin.

### 4.2. Description

Fossil seed cones are ovate, symmetric, 1.6–2.2 cm long and 1.2–1.6 cm wide, with a length-to-width ratio of 1.25–1.75. Base is broadly rounded, and apex is narrow rounded (Figure 2A–H). Each seed cone contains 19–27 cone-scale complexes in imbricate and are spirally arranged. Ovuliferous scales are rhombic or suborbicular in shape with a narrowed basal part, usually with rounded apex and auriculate bases in the middle part of scales (Figure 2K,J), 0.6–0.9 cm long and 0.5–0.7 cm wide, with the upper margin semicircular and contracted into a small, denticulate tip, and dwindle towards to the base auriculate. The abaxial surface of the scales is convex (Figure 2I,K,L), covered with several stripes; abaxial surface of the scale is striated (Figure 2G). Bracts are ligulate-spathulate in shape, 1/2 to 2/3 as long as seed scales and not covered by the lower ovuliferous scales (Figure 2A–H). There is a slight contraction in the middle of the bract (Figure 2L), apex acute or acuminate, with a dentate margin (Figure 2K,L).

## 5. Discussion

### 5.1. Comparisons with Extant Taxa Pinaceae

The seed cones of *Nothotsuga* mulaensis are characterized by ovate seed cones with 19–27 cone-scale complexes with imbricate and spiral arrangements, and the abaxial surface of the scales is covered with several stripes. Moreover, the ovuliferous scales are rhombic or suborbicular in shape with a narrowed basal part, a rounded apex, and auriculate bases in the middle part, and bracts are ligulate-spathulate in shape and usually exserted, 1/2 to 2/3 as long as seed scales. These morphological structures of seed cones are more or less similar to these genera, *Cathaya* Chun & Kuang, *Abies* Miller, *Keteleeria* Carr., *Larix* Miller, *Pseudotsuga* Carrière, *Tsuga* Carr., and Nothotsuga in the family Pinaceae (Table S1).

**Figure 3:**
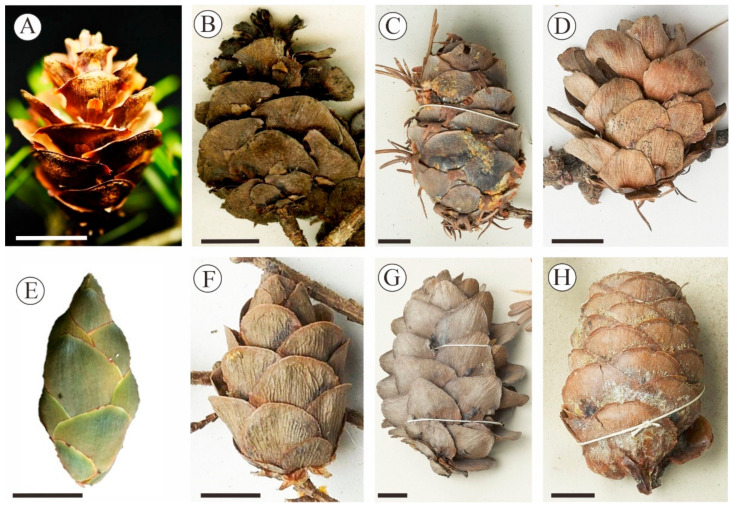
Morphological comparisons for the seed cones from some extant genera within the family Pinaceae. (**A**,**B**) *Nothotsuga longibracteata*. (**C**) *Pseudotsuga forrestii*. (**D**) *Larix sibirica*. (**E**) *Cathaya argyrophylla.* (**F**) *Tsuga dumosa*. (**G**) *Keteleeria davidiana*. (**H**) *Keteleeria fortunei*. Scale bars = 10 mm.

The monotypic genus *Cathaya* with a living species *C. argyrophylla* Chun & Kuang, is endemic to the mountain regions in central and southwest China, and has similar seed cones, with regard to shape, to the present fossil species. However, *C. argyrophylla* is characterized by 13–16 ovuliferous scales and triangular bracts that are not exserted [31,32], which significantly differ from the present fossil seed cones characterized by auriculate scales and exserted ligulate-spathulate bracts (Figure 2A–L). The bracts of *Abies* and *Keteleeria* are also exserted, which are similar to the present fossil species, whereas the seed cones of *Abies* and *Keteleeria* are significantly larger and have more cone-scale complexes than our fossil seed cones (Table S1). For example, the seed cones of *Abies fargesii* Franch are about 5.0–8.0 cm long and 3.0–4.0 cm wide, and those of *A. gorgei* Orr. is 7.0–11.0 cm long and 4.0–5.5 cm wide, and *K. davidiana* (Bertr.) Beissn is 8.0–21.0 cm long and 3.5–6.0 cm wide (Figure 3G) [29]. In addition, the scales of *Abies* are reniform or flabellate, and the scales of *Keteleeria* are ovate or rhombic, which distinctly differ from the broadly rhombic or suborbicular scales in our fossils [28,29]. Furthermore, the bracts of *A. fargesii* are obovate cuneate, with a rounded or slightly concave apex and central tips, and the bracts of *K. fortunei* (Murr.) Carr. (Figure 3H) are trilobate at the apex [17,29], which are obviously different from the ligulate-spathulate bracts with acute or acuminate apex in our fossils (Figure 2K).

**Figure 4:**
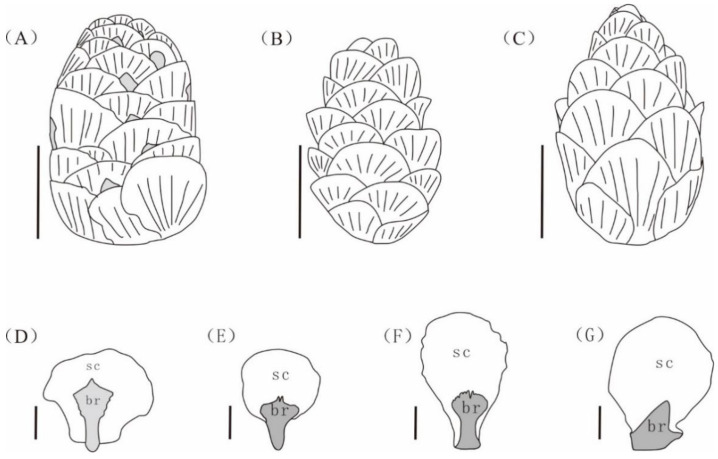
The line drawing of seed cones and bract-scale complexes of *Nothotsuga mulaensis* and extant *Tsuga*. br = bract, sc = scale. (**A**) Seed cone of *N. mulaensis*. (**B**) Seed cone of *T. chinensis*. (**C**) Seed cone of *T. dumosa.* (**D**) Scale-bract complexes of *N. mulaensis*. (**E**) Scale-bract complexes of *T. chinensis*. (**F**,**G**) Scale-bract complexes of *T. dumosa*. (**A**–**C**) Scale bar = 10 mm; (**D**–**G**) Scale bar = 5 mm.

The seed cones of *Larix* and *Pseudotsuga* are similar to present fossil seed cones in shape. Furthermore, elongate-spathulate bracts are also observed in the mature cones of *Larix*. However, the tips of the bracts are awned in *L. occidentalis* Nutt. and *L. lyallii* Parl, and both have more scales (45–55) [17], both of which are different from those of our fossil species. The seed cones of *L. mastersiana* Rehd. & Wils and *L. potaninii* Batalin both differ from our fossils by having long bracts with a reflexed apex [17,28,29]. The bracts of *Pseudotsuga* are also trilobate medium cracks with slightly reflexed and exserted bracts [17,28,29]. The fossil species is more similar to the genus *Tsuga* in shape and size of the seed cones, while the main difference is that the bracts are short and hidden behind the seed scales in *Tsuga*, and the bracts are exposed in the present fossil species [30,33]. In addition, the bracts of *Tsuga* are diverse, flabellate, trapezial, or cuneate (Figure 4E–G) [34,35], while the scales are rhombic to suborbicular with auriculate bases and the bracts are ligulate-spathulate in the present specimens.

*Nothotsuga* is a monotypic genus with only one species, *Nothotsuga longibracteata*. The auriculate scales and ligulate-spathulate bracts, which are exserted from the scales, are the most important features for an assignment to *Nothotsuga* [16,17,28,30,34]. The fossil seed cones described in this paper bear a striking resemblance to the seed cones of *N. longibracteata* (Figure 2I–L). According to the detailed morphological comparisons above, the fossil seed cones unequivocally classify into the genus *Nothotsuga*. However, *N. longibracteata* has more and smaller ovuliferous scales and larger seed cones than the present fossil species (Table S1). Furthermore, we find that *N. longibracteata* has smaller ovuliferous scales in the base than those in the middle (Figure 3A), while the scales are similar size in the base and in the middle part for *N. mulaensis*.

The fossil record of *Nothotsuga* is relatively sparse. To date, only two fossil species have been established based on fossil seed cones that were collected from the upper Miocene of eastern China and the Pliocene of Germany [17,31]. *Nothotsuga* vanderburghii (Gossmann ex Winterscheid & Gossmann) Xiaohui Xu, was reported in the lower Pliocene of Germany [32]. It was initially published as a fossil species of genus *Cathaya*, and later transferred into the *Nothotsuga* by its auriculate scales and slightly exserted ligulate-spathulate bracts, that are apically triangular and dentate by Xu et al. [31], which are in line with our fossil seed cones. However, *N. vanderburghii* has an ovate to conical seed cone with an acute apex and broad scales [31], which is different from those of our fossil seed cones.

*Nothotsuga sinogaia* were reported from the late Miocene, Zhejiang Province, eastern China, and are characterized by auriculate scales and subspathulate bracts. However, the seed cones of *N. sinogaia* are distinctly larger, ca. 6.0–6.9 cm long and 2.8–3.6 cm wide, than that of present specimens (1.6–2.2 cm long and 1.2–1.6 cm wide) [17]. Moreover, *N. sinogaia* has more ovuliferous scales and larger bracts (1.0–1.9 cm long and 1.7–2.5 cm wide) than the present fossil species (Table S1). Miki [36] treated several seed cones from the Pliocene of Japan as a separate fossil subgenus *Palaeotsuga*, and they differ from our specimens in the larger seed cones and linear long bracts. Therefore, our specimens are treated as a new fossil species *N. mulaensis* Z. Li & J. L. Dong sp. nov.

### 5.2. Implications for Biogeography of Nothotsuga

**Figure 5:**
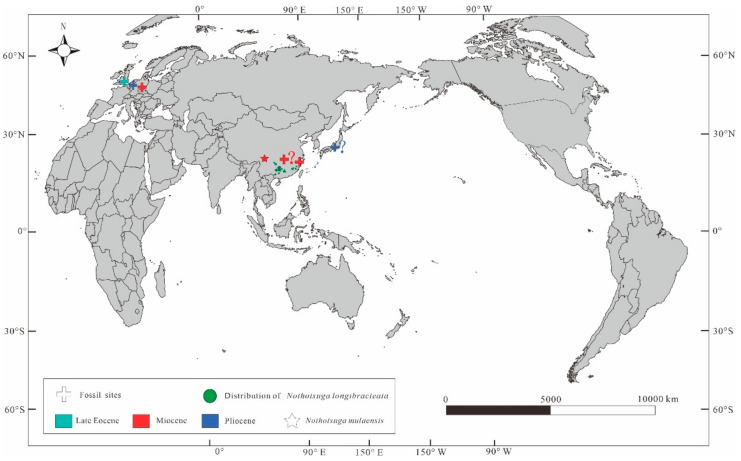
The spatial distribution map of fossil records and modern species of *Nothotsuga* [14,17,31,37].

*Nothotsuga* is well represented in the modern subtropical flora of southern China, including in northeastern Guizhou, southern Hunan, northern Guangdong, northeastern Guangxi, southern Fujian, and southern Jiangxi, China (Figure 5) [10,11]; however, the fossil record of this genus in these regions is poor. The reliable fossils are leaves from the late Eocene of Baltic amber [14] and from the early Miocene Wiesa flora of Saxony (Germany) [15,16]. Later, some cones were found from the late Miocene of the Shengxian Formation in Zhejiang [17] and from the lower Pliocene of Germany [31,32], and wood was reported from the Neogene Hubei [37]. The fossil seed cones of *Nothotsuga* from the Miocene in the eastern TP (examined in this study) have broadened the assumed spatial distribution of this species to the eastern TP and indicate that *Nothotsuga* once had a much broader distributional range and more northern distribution during the Miocene in China than they have today.

As mentioned above, *Nothotsuga* has a much earlier fossil history in Europe [14]. Nevertheless, it is still not possible to determine when this endemic genus appeared in China and finally achieved its endemic status; however, it is known that its sister genus, *Tsuga*, has been widely distributed in eastern Tibet since the late Eocene [30]. Ding et al. [17] hold that East Asia might be the center of origin for the *Nothotsuga*-*Tsuga* clade, based on their phylogenetic studies. Furthermore, our findings have emphasized that the eastern TP may play an important role in the dispersal and communication of *Nothotsuga* biodiversity.

### 5.3. Implications for the Paleoclimate and Paleoecology of Nothotsuga

Climate change, particularly temperature and precipitation, is the main factor restricting the distribution of *Nothotsuga*, as well as its species richness, and growth and development [11]. *Nothotsuga longibracteata* has a narrow range of habitat requirements and prefers abundant rainfall; it usually develops well with a mean annual precipitation of 1100–2000 mm, and a mean annual temperature (MAT) of 13.0–18.5 °C (Table 1) [8,11]. In addition, it grows in a mixed mesophytic forest with ‘subtropical’ and evergreen elements, in climate ranging from warm-temperate to ‘subtropical’ humid in southern China [11,14]. Therefore, we consider that the discovery of fossil *N. mulaensis* seed cones in Xiamula village, Litang County, eastern TP, might indicate that there was a warm and humid climate in this area during the Miocene.

The overall assemblage composition of the sediment layer in which the seed cones were found, included the aquatic plants *Trapa*, *Tsuga*, and *Rhododendron* [26]; all of which enjoy a warm and humid environment. This further indicates that Litang County had a shallow lake and a warm and humid environment during the Miocene [26,39]. Subsequent paleoclimatic changes, possibly as a response to the TP landscape’s evolution, affected the *Nothotsuga* population dynamics and their geographic distribution. *Nothotsuga* is highly sensitive to precipitation in the driest month and to the annual temperature range [17] and, thus, may respond very quickly to climate fluctuations. *Nothotsuga* possibly became extinct due to regional aridification and cooling [40], whereas its modern congeners are widely distributed in southern China.

However, the ancient topography of the eastern TP and its role in climatic and biotic evolution remain speculative due to sparse fossil records [41]. The extant *Nothotsuga* is mostly distributed at altitudes of 300–2300 m, and the aquatic plant *Trapa* also has similar ecological requirements, and it is not found at altitudes higher than 2300 m [42]. This suggests that the area might not have reached its present elevation, ca. 3700 m (Table 1), during the Miocene. However, newly discovered fossils from the Relu Basin and Mangkang Basin show that the eastern TP had reached its current elevation during the late Eocene, with altitudes ranging from 3000 to 3500 m [43,44]. This suggests that there may have been an intermountain lake basin in the area. In Xiamula village, the *Nothotsuga* cone-bearing layer of the Changtai Formation is rich in both mixed evergreen forest and alpine shrub forest, indicating a coniferous forest landscape and a mixed alpine shrub forest, which was dominated by *Tsuga*, *Picea*, *Rhododendron,* and *Abies*, with altitudes of 2000–3500 m [26]. In summary, we consider the paleoelevation of the intermountain lake basin in Xiamula village to be 2000–2300 m, and the surrounding mountains may have reached 3500 m [44]. The warm and humid intermountain basin would have provided a great ecological environment for subtropical coniferous forests.

## 6. Conclusions

A new fossil species, *Nothotsuga* mulaensis, was reported based on seed cones discovered in the Mula Basin, Litang County, Sichuan Province. The fossil species required humid subtropical vegetation, as suggested by its nearest living relative *N. longibracteata*. This discovery suggests that the Mula region had a warm and humid climate during the Miocene. We propose that the climate change that occurred after the Miocene caused the disappearance of *Nothotsuga* in the eastern TP. There was an intermountain lake basin at the site of this discovery, which was surrounded by high mountains, at an altitude of 3500 m. Our findings have highlighted the complexity of the Tibetan Plateau’s ancient landscape and have emphasized the importance of the Tibetan Plateau in the history of *Nothotsuga* biodiversity.

## Figures and Tables

**Table 1:** The present climatic parameters of fossil localities and climate ranges of *Nothotsuga longibracteata*. Modern values are from the Litang meteorological station.

Site/Species	Mean Annual Temperature MAT (°C)	Coldest Monthly Mean Temperature (CMMT) (°C)	Mean Temperature of the Warmest Month (MTWM) (°C)	Annual Range of Monthly Mean Temperature (ART) (°C)	Mean Annual Precipitation (MAP) (mm)	Altitude (m)	Reference
*Nothotsuga longibracteata*	13.0–18.5	2.0–9.0	21.0–28.5	18.0–20.0	1100–2000	300–2300	[8,11,12,38]
Modern Litang Country	5.7	−5.35	12.1	18.0	741.6	3700	This paper

## Data Availability

All data generated by this study are available in this manuscript and the accompanying Supplementary Materials.

## Supplementary Materials

The following supporting information can be downloaded at https://www.mdpi.com/article/10.3390/biology12010046/s1; Table S1: Comparisons of seed cones of *Nothotsuga mulaensis* Z. Li & J.L. Dong with fossil species of *Nothotsuga* and other similar taxa of Pinaceae [45,46].
