# Supplementary material for: A New Fossil Species of Nothotsuga from the Mula Basin, Litang County, Sichuan Province and Its Paleoclimate and Paleoecology Significance
Source: Biology (Basel). 2022 Dec 26;12(1):46. doi: 10.3390/biology12010046 (PMC9855038; doi:10.3390/biology12010046)
Supplement: Supplementary file 1 [file biology-12-00046-s001.zip › biology-2080505-supplementary.pdf]

Table S1. Comparisons of seed cones of *Nothotsuga mulaensis* Z. Li et J.L. Dong with fossil species of *Nothotsuga* and other similar taxa of Pinaceae [1–10].

**Taxa**	**age**	**Seed cone shape**	**Seed cone length×width (cm)**	**Scale number**	**Scale shape**	**Scale apex**	**Scale length×width (cm)**	**Bract shape**	**Bract form**	**Bract apex**
Fossil species										
Nothotsuga mulaensis	Miocene	Ovate	1.6–2.2 × 1.2–1.6	19–27	Rhombic or suborbicular	Rounded	0.6–0.9 × 0.5–0.7	Ligulate-spathulate	Exserted	Acute or acuminate
Nothotsuga sinogaia	Miocene	Ovate to elliptic	5.8–7.2 × 3.6–3.6	26–32	Broadly rhombic	Truncate-rounded	1.0–1.9 × 1.7–2.5	Subspatulate	Exserted	Acute or acuminate
Nothotsuga vanderburghii	Pliocene to Pleistocene	Ovate to conical	1.4–4.5 × 0.9–1.9	20	Broadly rhombic or suborbicular	Semicircular	0.5–1.2 × 0.7–1.8	Ligulate-spathulate	Slight exserted	Triangular
Nothotsuga longibracteata	Pliocene	Ovate or oblong-ovate	2.0–2.7 × 1.0–1.3		Elliptic	–	1.0–1.5× 1.0–1.3	Linear	–	–
Extant species										
Nothotsuga longibracteata	–	Ovate to elliptic	2.0–5.8 × 1.2–2.5	24–36	Broadly rhombic or suborbicular	Truncate-rounded	0.9–2.2 × 1.2–2.5	Subspatulate	Exserted	Acute or acuminate
Tsuga dumosa	–	Ovate	1.5–3.0 × 1.0–2.0	30	Rectangular rounded, obovate rectangular rounded or ovate-oblong	Broad rounded or truncate	1.0–1.4 × 0.7–1.2	Trapezial or cuneate	Hidden	Round
Cathaya argyrophylla	–	Ovate to elliptic	3.0–5.0 × 1.5–3.0	13–16	Suborbicular or ovate	Rounded	1.5–2.5 × 1.0–2.5	Triangular	Hidden	Acute

Keteleeria davidiana	–	Cylindric or ovoid-cylindric	8.0–21.0 × 3.5–6.0	65–90	Narrowing to the rounded	Subacute	2.6–3.2 × 2.2–2.8	Spatulate	Exserted	Trilobate
Keteleeria evelyniana	–	Cylindric	9.0–20.0 × 4.0–6.5	68–102	Rhombic-ovate	Subacute	3.0–4.0 × 2.5–3.0	Spatulate	Exserted or hidden	Trilobate
Keteleeria fortunei	–	Cylindric or oblong-cylindric	6.0–18.0 × 5.0–6.5	72–97	Orbicular, rhombicorbicular	Rounded, middle concave	2.5–3.2 × 2.7–3.3	Spatulate	Exserted or hidden	Trilobate
Pseudotsuga brevifolia	–	Ovate-ellipsoid or ovate	3.7–6.5 × 3.0–4.0	24–38	Orbicular-rhombic	Broad rounded	2.2–2.5 × 3.3	Spatulate	Exserted	Trilobate, reflexed
Pseudotsuga forrestii	–	Ovate	5.8 × 4.0–5.5	30–40	Suborbicular or rhombic-rounded	Truncated-rounded	2.5–3.5 × 3.0–4.0	Spatulate	Exserted	Trilobate, reflexed
Pseudotsuga japonica	–	Ovate or oblong-ovate	4.0–5.0 × 2.0–2.5	15–20	Broadly cuneate-flabellate	Rounded	1.5–1.8 × 2.0–2.5	Spatulate	Exserted	Trilobate, reflexed
Pseudotsuga macrocarpa	–	Ovate-cylindric	12.0–15.0 × 4.0–6.0	24–35	Cuneate-flabellate	Rounded	–	Ligulate	Exserted	Trilobate
Pseudotsuga menziesii	–	Ovate	8.0 × 3.5–4.0	26–40	Cuneate-nearly rhombic	Rounded	–	Ligulate	Exserted	Trilobate
Pseudotsuga sinensis	–	Ovate to ellipsoidal or conical-ovate	4.5–8.0 × 3.5–4.5	24–38	Semiorbicular	Rounded	2.5 × 3.0	Ligulate	Exserted	Trilobate, reflexed
Larix gmelinii	–	Ovate-oblong	1.2–3.0 × 1.0–2.0	14–30	Pentagonal-ovate	Rounded or truncate	1.0–1.5 × 0.8–1.2	Broad ligulate–lanceolate	Hidden	Trilobate
Larix griffithii	–	Cylindric or cylindric-ellipsoid	5.0–11.0 × 2.2–3.0	50–100	Obovate-square	Truncate or slightly emarginate	1.4 × 1.2	Ovate–or obovate–lanceolate	Exserted	Trilobate, reflexed

Larix kaempferi	–	Broadly ovate	2.0–3.5 × 1.8–2.8	46–65	Suborbicular	Truncate or slightly emarginate	1.2–1.5 × 1.0	Ligulate	Hidden	Acuminate
Larix laricina	–	Ovate	1.0–2.0 × 0.5–1.0	10–30	Suborbicular	Entire	–	–	Hidden	Mucronate or tipped by awn
Larix lyallii	–	Elliptic	2.5–4.0 × 1.1–1.9	45–55	Rounded	Entire	–	Ligulate	Exserted	Tipped by awn
Larix mastersiana	–	Cylindric-ellipsoid	2.5–4.0 × 1.5–2.0	45–80	Obovate-reniform	Entire, emarginate	0.8–1.1 × 1.0–1.3	Broadly lanceolate	Exserted	Reflexed
Larix occidentalis	–	Ovate	2.0–3.0 × 1.3–1.6	45–55	-	Entire	–	Ligulate	Exserted	Tipped by awn
Larix potaninii	–	Cylindric to ovate	3.0–5.0 × 1.5–2.5	35–65	Subquadrate or squoval	Obtuse or notched	0.8–1.3 × 0.8–1.1	Oblong–lanceolate	Exserted	Acute or acuminate
Larix sibirica	–	Ovate	2.0–4.0 × 1.5–3.0	25–40	Ovate or narrowly rhombic–ovate	Rounded	1.5–1.8 × 1.0–1.4	Oblong–lanceolate	Exserted	Midvein Elongated into a caudate cusp
Abies fargesii	–	Cylindrical rectangular circle or Cylindric	5.0–8.0 × 3.0–4.0	130	Reniform-flabellate	Rounded	0.8–1.2 × 1.5–2.0	Obovate cuneate	Slight exserted	Acute or acuminate
Abies nephrolepis	–	Ovate cylinder or cylindroid	4.5–9.5 × 2.0–3.0	200	Reniform-flabellate	Broad rounded	1.0–1.5 × 1.4–2.2	Obovate	Slight exserted	Emarginate, or acute
Abies georgei	–	Ovate cylinder	7.0–11.0 × 4.0–5.5	275	Flabellate-squared	Broad rounded	1.9–2.1 × 1.8–2.3	Spatulate	Slight exserted	Triangular

References

1. Ding, L.; Spicer, R.A.; Yang, J.; Xu, Q.; Cai, F.L.; Li, S.; Lai, Q.Z.; Wang, H.Q.; Spicer, T.; Yue, Y.H. Quantifying the rise of the Himalaya orogen and implications for the South Asian monsoon. *Geology* **2017**, *45*: 215–218.
2. Ding, S.T.; Chen, S.Y.; Ruan, S.C.; Yang, M.; Han, Y.; Wang, X.H.; Zhang, T.H.; Sun, B.N. First fossil record of *Nothotsuga* (Pinaceae) in China: implications for palaeobiogeography and palaeoecology. *Hist. Biol.* **2021**, *33*, 3617–3624.
3. Karavayev, M.N. *Tsuga longibracteata* Cheng, first found in a fossil condition on the territory of the USSR. *Bulletin de la Société des Naturalistes de Moscou, Section Biologique* **1958**, 63, 73–76.
4. Xu, X.H.; Zhang, X.; Yang, L.Y. *Cathaya vanderburghii*, a misnomer for European Neogene fossil cones. *Taxon* **2022**, 71, 1107–1111.
5. Miki, S. The occurrence of the remain of *Taiwania* and *Palaeotsuga* (n. subg.) from Pliocene beds in Japan. *Proc. Jpn. Acad.* **1954**, *30(10)*: 976–981.
6. Mai, D.H. Fossile Koniferenreste in der meridionalen Zone Europas. Feddes Repert **1994**, 105: 207–227.
7. Martinetto, E. *East Asian elements in the Plio-Pleistocene floras of Italy*. pp. 71–87 in: Zhang, A.L.&Wu, S.G. (eds.), *Proceedings of the First International Symposium on Floristic Characteristics and Diversity of East Asian Plants*, 25–27 July 1996, Kunming, Yunnan, P.R. China. Berlin: Springer. 1998.
8. Winterscheid, H., Gossmann, R. Validation of Cathaya vanderburghii (Pinaceae) from European Neogene. *Phytotaxa* **2017**, *302*: 188–192.
9. Fu, L., K, Li, N., Elias, T.S., Mill, R.R. Pinaceae. In: Wu Z.Y. editor. *Flora of China* (Vol. 4). Beijing & St. Louis: Science Press & Missouri Botanical Garden Press 1999, 11–52.
10. Earle, C.J. Gymnosperm database. http://www.conifers.org/ **2020**.
